# On the Storage–Communication Trade-Off in Graph-Based *X*-Secure *T*-Private Linear Computation

**DOI:** 10.3390/e27090975

**Published:** 2025-09-18

**Authors:** Yueyang Liu, Haobo Jia, Zhuqing Jia

**Affiliations:** School of Artificial Intelligence, Beijing University of Posts and Telecommunications, Beijing 100876, China; liuyueyang@bupt.edu.cn (Y.L.); jiahaobo@bupt.edu.cn (H.J.)

**Keywords:** linear computation, communication efficiency, storage efficiency, cross subspace alignment, private information retrieval

## Abstract

The problem of graph-based *X*-secure *T*-private linear computation (GXSTPLC) is to allow a user to retrieve a linear combination of *K* messages from a set of *N* distributed servers that store the messages in a graph-based fashion, i.e., each message is restricted to be distributed among a subset of servers. *T*-privacy requires that the coefficients of the linear combination are not revealed to any group of up to *T* colluding servers, and *X*-security guarantees that any set of up to *X* colluding servers learns nothing about the messages. In this paper, we propose an achievability scheme for GXSTPLC that enables a storage–communication trade-off by exploiting non-replicated storage codes. Novel aspects of our achievability scheme include the usage of the idea of cross-subspace alignment null shaper that addresses various challenges posed by the graph-based storage structure. In addition, unlike previous works, our scheme allows a direct transformation into a quantum one to achieve a superdense coding gain by leveraging the idea of *N*-Sum Box abstraction of quantum “over-the-air” computing.

## 1. Introduction

Escalating concerns regarding security and privacy in distributed systems motivate the problem of private linear computation (PLC). PLC considers a scenario where *K* messages are stored (possibly replicated or coded) across *N* distributed servers. The user aims to retrieve a linear combination of these messages without revealing the coefficients of the linear combination to any group of up to *T* colluding servers, where *T*, representing the maximum number of tolerable colluding servers, is referred to as the privacy threshold. Notably, PLC is a non-trivial generalization of private information retrieval (PIR), as PIR corresponds to the special case where the user uses a one-hot coefficient vector to retrieve a single message. Recent advancements in the study of PIR and PLC from an information-theoretic perspective have yielded a series of capacity (i.e., the reciprocal of the minimum possible normalized download cost across *N* servers) characterizations and novel coding schemes for these problems and their variants [1,2,3,4,5,6,7,8,9,10,11,12,13,14,15,16,17,18,19,20,21,22,23,24,25,26,27,28,29,30,31,32,33,34,35,36,37,38,39,40,41,42,43,44,45].

Existing paradigms like PIR and PLC often assume global data availability, where each message can be stored across all servers, which is cumbersome in real-world applications due to constraints such as geographic blocking, network connectivity, and data security, so that data are not uniformly available at all servers, and data must be secure against storage eavesdroppers. Driven by this problem, a PLC variant known as graph-based *X*-secure *T*-private linear computation (GXSTPLC) incorporates two key constraints. First, the non-uniform data availability, or graph-based PLC, where each message is restricted to be stored among a specific subset of servers, referred to as the storage pattern. This pattern can be naturally represented by a hypergraph where servers are nodes and the storage subset for each message forms a hyperedge. Second, *X*-security of the storage, a standard requirement ensuring that no information about the messages is revealed to any set of up to *X* colluding servers. While several prior works address graph-based PIR/PLC [35,36,37,38], and the asymptotic (i.e., in the limit as the number of messages *K* approaches infinity) capacity of GXSTPLC was fully characterized in [38], a subtle observation arises. To the best of our knowledge, the storage codes in related prior graph-based PIR/PLC works are essentially repetition codes (meaning each stored portion of a message is at least as large as the original message). Furthermore, the achievability scheme in [38] may necessitate storage exceeding simple replication, which can be highly storage-inefficient. Our work is motivated by this potential storage inefficiency; i.e., we are interested in the trade-off between the download cost and storage efficiency by leveraging coding techniques in the storage construction. We note that deriving the tight converse bound (i.e., the full capacity characterization) for the storage–download trade-off of GXSTPLC remains a challenging open problem. The focus of this work is to establish the first achievability scheme that enables such a trade-off.

The main result of this work is an achievability scheme for GXSTPLC that allows a trade-off between the download cost and storage cost. Specifically, we first propose an achievability scheme for a closely related problem, namely Asymmetric MDS-GXSTPLC, where security and privacy requirements are non-uniform across the messages; i.e., each message and the corresponding coefficient are specified with individual security and privacy thresholds. In addition, the storage code for each message is an MDS code that results in a reduction in storage cost (by trading off the communication efficiency). Then, based on the achievability scheme for Asymmetric MDS-GXSTPLC, our achievability scheme is finalized by adapting the idea of the augmented system in [38]. The key novelty that distinguishes our scheme from previous state-of-the-art is summarized as follows:

1. The idea of exploiting MDS codes for the storage in graph-based PIR/PLC: Ref. [38] achieves a single point (minimum download cost) using replication codes that turn out to be storage-inefficient, while our scheme leverages MDS-coded storage to allow a storage–download trade-off. To the best of our knowledge, this is the first scheme to incorporate MDS codes in graph-based PIR/PLC.
2. The technique used to handle the challenges introduced by the graph-based storage structure: Our work introduces a novel technique centered around the idea of a cross-subspace alignment (CSA) null shaper introduced in [27] to address the challenges introduced by the graph-based storage structure. The CSA null shaper was originally designed for storage-consistent private updates with unavailable servers. However, in this work, this idea is adapted to ensure that the overall storage conforms to valid CSA codewords under the graph-based storage constraints. This distinguishes our scheme from the scheme in [38], where the PLC under graph-based storage structure is enabled by a combination of techniques including CSA codes, dual Generalized Reed–Solomon (GRS) codes, and a Vandermonde decomposition of Cauchy matrices. Intuitively, CSA codes can be viewed as evaluation codes, and the CSA null shaper carefully places zeros at certain evaluation points, which correspond precisely to the servers prohibited by the graph-based storage pattern from storing codewords of a particular message. Consequently, the codewords for these servers are explicitly set to zero, requiring no storage at all, and the overall codewords (including zeros) remain valid CSA codewords. It should be noted that the idea of placing zeros in the storage construction for graph-based PIR/PLC may be profound, as the storage code of many known PIR/PLC schemes can be viewed as evaluation codes (e.g., polynomial codes based PIR/PLC in [12,24,46,47,48,49]). This idea may transform known PIR/PLC schemes into graph-based ones.
3. Reduced decoding complexity and quantum adaptability: Unlike schemes based on dual GRS codes properties, where a **pre-processing step** of interference cancellation during decoding is generally necessary, in our scheme, the user can recover the desired linear combination by merely solving linear systems defined by Cauchy–Vandermonde matrices, hence the reduction in decoding complexity. Moreover, our scheme is compatible with the *N*-Sum Box abstraction of quantum “over-the-air” computing [44,50], enabling a direct transformation of our scheme into a quantum one to achieve the superdense coding gain.

*Notations:* $Z_{>0}$ denotes the set of positive integers. The set of rational numbers is denoted as Q, while $Q_{\geq 0}$ denotes the set of *non-negative* rational numbers. For any two positive integers M<N, [M:N] denotes the set {M,M+1,⋯,N}, and [N] denotes [1:N]. For any set of random variables $X_{1},X_{2},\cdots ,X_{N}$ indexed by [N], and an index set I⊂[N], $X_{I}$ denotes $\left\{X_{i}|i\in I\right\}$. For any x∈R, ${\left(x\right)}^{+}$ denotes max(x,0).

## 2. Problem Statement

**Figure 1:**
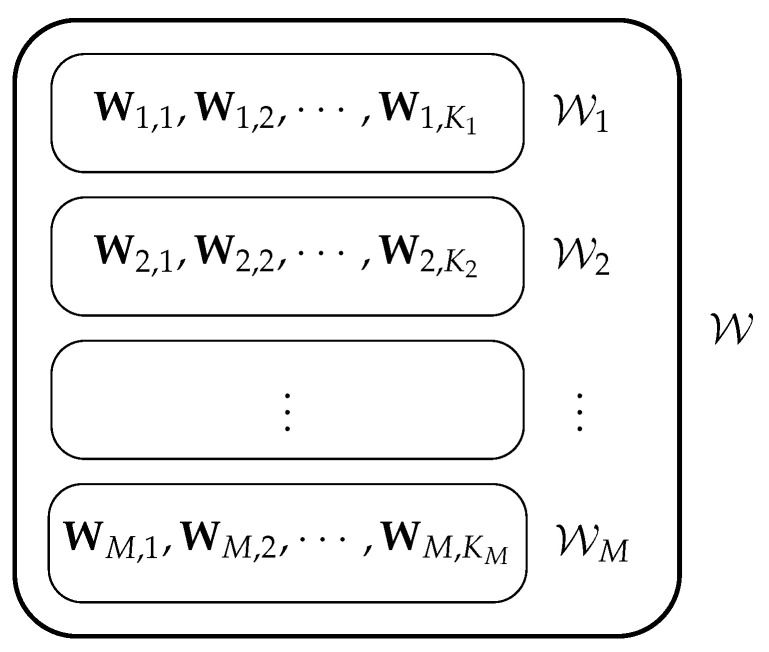
The *K* messages are partitioned into *M* disjoint message sets, where the *m*-th message set consists of $K_{m}$ messages.

Let us consider a private linear computation problem with *N* servers, denoted as Server n,n∈[N], and *K* messages. As depicted in Figure 1, the messages are partitioned into *M* disjoint sets, i.e., $W={⋃}_{m\in [M]}W_{m},W_{i}\cap W_{j}=\emptyset ,\;\forall i\neq j,\;i,j\in \left[M\right]$, where $W_{m}$ is the $m^{th}$ message set and W is the set of all messages. For each m∈[M], we define $W_{m}=\left\{W_{m,1},W_{m,2},\cdots ,W_{m,K_{m}}\right\}$; i.e., the message set $W_{m}$ consists of $K_{m}$ messages, and each of the messages comprises *L* i.i.d. symbols from a finite field $F_{q}$. Formally, for all $m\in \left[m\right],k\in \left[K_{m}\right]$, we have $W_{m,k}={\left[W_{m,k}\left(1\right),W_{m,k}\left(2\right),\cdots ,W_{m,k}\left(L\right)\right]}^{⊤}$, and

(1) $$\begin{array}{c} H({\left(W_{m,k}\right)}_{m\in \left[M\right],k\in \left[K_{m}\right]})=KL, \end{array}$$

in *q*-ary units. Due to non-uniform data availability, each message set is only allowed to be stored among a subset of the *N* servers. To characterize this graph-based storage fashion, let us define

(2) $$\begin{array}{cc} R & =\{R_{1},R_{2},\cdots ,R_{M}\}, \end{array}$$

(3) $$\begin{array}{cc} R_{m} & =\left\{R_{m}\left(1\right),R_{m}\left(2\right),\cdots ,R_{m}\left(\rho_{m}\right)\right\}\subset \left[N\right], \end{array}$$

where $R_{m}$ corresponds to the $m^{th}$ message set $W_{m}$, and for all m∈[M], $R_{m}$ represents a subset of servers; i.e., the message set $W_{m}$ is allowed to be stored among Server $n,n\in R_{m}$. The collection of server subsets R is referred to as the storage pattern. Note that it is occasionally more convenient to consider the dual representation of the storage pattern,

(4) $$\begin{array}{cc} M & =\{M_{1},M_{2},\cdots ,M_{N}\}, \end{array}$$

(5) $$\begin{array}{cc} M_{n} & =\{m\in \left[M\right]|R_{m}∋n\}, \end{array}$$

i.e., for all n∈[N], Server *n* is allowed to store a securely coded codeword of the messages $W_{m},m\in M_{n}$. In other words, denoting the codeword of the message $W_{m,k},m\in \left[M\right],$$k\in [K_{m}]$ stored at Server $n,n\in R_{m}$ as ${\tilde{W}}_{m,k}^{\left(n\right)}$, the storage at server n,n∈[N], denoted as $S_{n}$, is, thus,

(6) $$\begin{array}{c} S_{n}=\left\{{\tilde{W}}_{m,k}^{\left(n\right)}|\;m\in M_{n},\;k\in \left[K_{m}\right]\right\}. \end{array}$$

Moreover, the *X*-secure storage constraint guarantees that the collusion of any up to *X* servers discloses nothing about the message, i.e.,

(7) $$\begin{array}{c} I\left(S_{X};W\right)=0,\;\forall X\subset \left[N\right],\;\left|X\right|=X. \end{array}$$

Let us further elaborate on the above notations via the following example. Assume that we have M=4 message sets $W_{1},W_{2},W_{3},W_{4}$ that are stored at N=7 servers, as shown in the following table.
Server 1Server 2Server 3Server 4Server 5Server 6Server 7Server 8$W_{2}$$W_{1}$$W_{1}$$W_{2}$$W_{2}$$W_{1}$$W_{1},W_{2}$$W_{1}$

For this example, we have

(8a) $$\begin{array}{cccccccc} R_{1} & =\{2,3,6,7\}, & \rho_{1} & =4 & R_{2} & =\{1,4,5,7,8\}, & \rho_{2} & =5 \end{array}$$

(8b) $$\begin{array}{cccccccc} M_{1} & =\{2\}, & M_{2} & =\{1\}, & M_{3} & =\{1\}, & M_{4} & =\{2\}, \end{array}$$

(8c) $$\begin{array}{cccccccc} M_{5} & =\{2\}, & M_{6} & =\{1\}, & M_{7} & =\{1,2\}, & M_{8} & =\{1\}. \end{array}$$

In our private linear computation problem, the user is interested in a linear combination of the *K* messages of the following form

(9) $$\begin{array}{c} \lambda_{\Lambda }\left(W\right)≜\sum_{m\in [M]}\sum_{k\in [K_{m}]}\lambda_{m,k}W_{m,k}, \end{array}$$

where $\Lambda ={\left(\lambda_{m,k}\right)}_{m\in \left[M\right],k\in \left[K_{m}\right]}$ is the coefficient of the linear combination and $\lambda_{m,k,l}\in F_{q},m\in \left[M\right],k\in \left[K_{m}\right],l\in \left[L\right]$ are generated by the user privately, uniformly i.i.d. over the finite field $F_{q}$. For this purpose, the user generates a total of *N* queries, $Q_{n}^{\left(\Lambda \right)},n\in \left[N\right]$, where $Q_{n}^{\left(\Lambda \right)}$ is intended for Server *n*, without prior awareness of messages or server storage

(10) $$\begin{array}{c} I(S_{\left[N\right]};\Lambda ,Q_{1}^{\left(\Lambda \right)},\cdots ,Q_{n}^{\left(\Lambda \right)})=0, \end{array}$$

so that any set of up to *T* colluding servers learns nothing about the coefficients Λ. Formally, we have

(11) $$\begin{array}{c} I\left(Q_{T}^{\left(\Lambda \right)};\Lambda \right)=0,\;\forall T\subset \left[N\right],\;\left|T\right|=T. \end{array}$$

Once the query $Q_{n}^{\left(\Lambda \right)}$ is available at Server *n*, n∈[N], an answer string $A_{n}^{\left(\Lambda \right)}$ is generated as a function of $Q_{n}^{\left(\Lambda \right)}$ and its storage $S_{n}$, i.e.,

(12) $$\begin{array}{c} H(A_{n}^{\left(\Lambda \right)}|Q_{n}^{\left(\Lambda \right)},S_{n})=0. \end{array}$$

A private linear computation scheme is correct if and only if the desired linear combination is resolvable from the collection of downloaded server answers, i.e.,

(13) $$\begin{array}{c} H(\lambda_{\Lambda }\left(W\right)|A_{\left[N\right]}^{\left(\Lambda \right)},Q_{\left[N\right]}^{\left(\Lambda \right)},\Lambda )=0. \end{array}$$

To characterize the communication efficiency of a private linear computation scheme per server, we define the normalized total download cost $D_{n}$ as the expected number of *q*-ary symbols downloaded from the *n*-th server, normalized by *L*, that is, $D_{n}=\frac{H(A_{n}^{\left(\Lambda \right)})}{L}$. In addition, to measure the storage efficiency per server, we define the normalized storage cost for all n∈[N] as follows:

(14) $$\begin{array}{c} C_{n}=\frac{H(S_{n})}{KL}. \end{array}$$

## 3. Main Result

The main result of this work is an achievability scheme for the problem of GXSTPLC that allows a trade-off between the download cost and storage cost, formally presented in the following theorem.

**Theorem** **1.** *Let $\eta =\left(\eta_{1},\eta_{2},\cdots ,\eta_{M}\right)\in Q^{M}$ be a vector of rational numbers such that for all $m\in \left[M\right],\eta_{m}\geq 1$, and each $\eta_{m}=p_{m}^{\prime }/q_{m}^{\prime }$, with $p_{m}^{\prime },q_{m}^{\prime }$ being co-prime. Consider a target vector of per-server normalized download costs $\left(D_{1},D_{2},\cdots ,D_{N}\right)\in Q_{\geq 0}^{N}$, where each $D_{n}=p_{n}/q_{n}$, with $p_{n},q_{n}$ being co-prime. If the vector $\left(D_{1},D_{2},\cdots ,D_{N}\right)$ lies within the achievable region D, defined as*

(15) $$\begin{array}{c} D\left(\eta \right)≜\left\{\left(D_{1},\cdots ,D_{N}\right)\in Q_{\geq 0}^{N}\left|\begin{array}{c} \forall m\in \left[M\right],\forall R_{m}^{\prime }\subseteq R_{m}\;\mathrm{with}\;\\ |R_{m}^{\prime }\left|=\right|R_{m}|-X-T,\sum_{n\in R_{m}^{\prime }}D_{n}\geq \eta_{m} \end{array}\right.\right\}, \end{array}$$

*then this vector of download costs is achievable by the proposed GXSTPLC scheme. The corresponding normalized storage cost $C_{n},n\in \left[N\right]$ is given by*

(16) $$\begin{array}{c} C_{n}=\frac{1}{K}\sum_{m\in M_{n}}\min\left(\tau_{n},\gamma_{m}\right)\frac{K_{m}}{q_{0}\left(\eta_{m}-1\right)+1}, \end{array}$$

*where the integers $\tau_{n}$ are defined as $\tau_{n}=q_{0}D_{n}$ with $q_{0}=lcm\left(q_{1},q_{2},\cdots ,q_{N},q_{1}^{\prime },q_{2}^{\prime },\cdots ,q_{M}^{\prime }\right)$, and for each m∈[M], $\gamma_{m}$ is the (X+T)-th largest value in the multiset $\left\{\tau_{n}|n\in R_{m}\right\}$ when its elements are sorted in non-increasing order.*

**Remark** **1.** *It is remarkable that by setting $\eta_{m}=1$ for all m∈[M], our result reduces to that of [38], which is capacity-achievable; i.e., it achieves the minimum possible normalized total download cost $D=\sum_{n\in [N]}D_{n}$ via the optimal configuration of $D_{n}$s. It is also worth mentioning that by setting $\eta_{m}=1$ for all m∈[M], the closure of our achievable region D(1) in the real space $R^{N}$ exactly matches the converse region established in [38] (Theorem 1).*

### The Storage–Communication Trade-Off in the Proposed GXSTPLC Scheme

In this section, we investigate the fundamental trade-off between the normalized storage cost $C_{n}$ and the normalized download cost $D_{n}$ of our proposed GXSTPLC scheme. To facilitate this analysis, we can bound the expression for $C_{n}$ as follows.

(17) $$\begin{array}{cc} C_{n} & =\frac{1}{K}\sum_{m\in M_{n}}\min\left(\tau_{n},\gamma_{m}\right)\frac{K_{m}}{q_{0}\left(\eta_{m}-1\right)+1} \end{array}$$

(18) $$\begin{array}{cc} \; & \leq \frac{1}{K}\sum_{m\in M_{n}}\tau_{n}\frac{K_{m}}{q_{0}\left(\eta_{m}-1+\frac{1}{q_{0}}\right)} \end{array}$$

(19) $$\begin{array}{cc} \; & =\frac{D_{n}}{K}\sum_{m\in M_{n}}\frac{K_{m}}{\eta_{m}-1+\frac{1}{q_{0}}}, \end{array}$$

where (18) holds by noting that $\min\left(\tau_{n},\gamma_{m}\right)\leq \tau_{n}$. To further simplify our analysis, let us consider a symmetric scenario where $K_{m}=K/M$ for all m∈[M]. In addition, by definition, $q_{0}$ tends to be large, so the bound can then be approximated as

(20) $$\begin{array}{c} C_{n}⪅\frac{D_{n}}{M}\sum_{m\in M_{n}}\frac{1}{\eta_{m}-1}. \end{array}$$

This approximation explicitly shows that for operating points within the achievable region D(η), for a given server *n*, its storage cost $C_{n}$ is directly proportional to its own download cost $D_{n}$ and inversely related to the term $\eta_{m}-1$ for each message set *m* it stores. This relationship suggests that one potential strategy to reduce $C_{n}$ is to increase the corresponding values of $\eta_{m}$ while keeping $D_{n}$ fixed. However, according to the definition of the achievable region D(η), increasing any $\eta_{m}$ tightens the feasibility constraint associated with the message set *m*, requiring a larger sum of download costs from the relevant servers. To satisfy this stricter condition, the download costs of other servers (i.e., those in $R_{m}$) must collectively increase. This increase in another server’s download cost, namely $D_{n^{\prime }}$, is likely to elevate its own storage cost $C_{n^{\prime }}$. Therefore, an attempt to locally optimize storage cost on one server can inadvertently shift the burden, increasing both download and storage costs elsewhere in the system.

On the other hand, it is also of interest to explore the trade-off between the total normalized storage cost, denoted by $C=\sum_{n=1}^{N}C_{n}$, and the total normalized download cost, $D=\sum_{n=1}^{N}D_{n}$. To construct the optimal trade-off curve of *D* versus *C* for a given total download cost $D_{\mathrm{target}}$, our goal is to find the minimum possible total storage cost $C_{\min}$. This is formulated as an optimization problem:

(21) $$\begin{array}{cc} C_{\min}\left(D_{\mathrm{target}}\right)=\min_{\eta ,{\left(D_{n}\right)}_{n\in [N]}} & \;\sum_{n=1}^{N}C_{n} \end{array}$$

(22) $$\begin{array}{cc} s.t.\; & \;\sum_{n=1}^{N}D_{n}\leq D_{\mathrm{target}}, \end{array}$$

(23) $$\begin{array}{cc} \; & \;\left(D_{1},D_{2},\cdots ,D_{N}\right)\in D\left(\eta \right), \end{array}$$

(24) $$\begin{array}{cc} \; & \;\eta_{m}\geq 1,\;\forall m\in \left[M\right]. \end{array}$$

**Figure 2:**
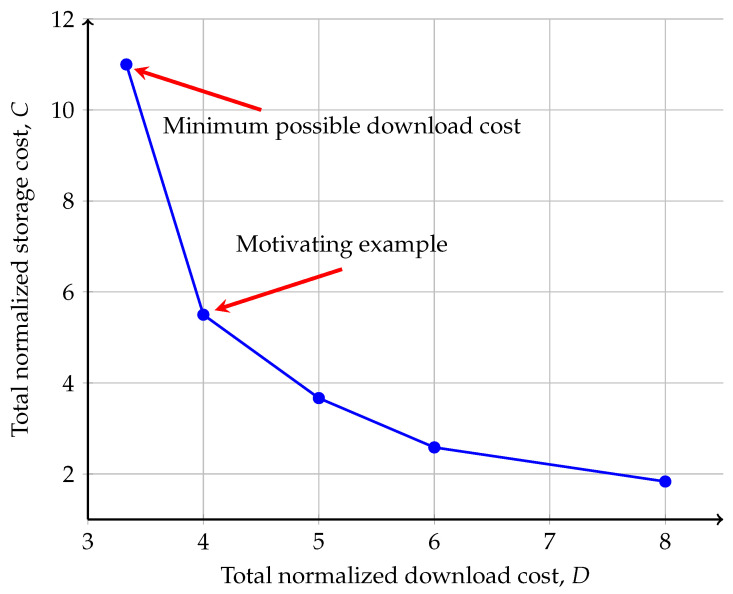
An illustrating example of the storage–communication trade-off in the proposed GXSTPLC scheme, where $N=8,X=1,T=1,K/K_{m}=M=2$, and the storage pattern is given by $R_{1}=\left\{2,3,6,7\right\},R_{2}=\left\{1,4,5,7,8\right\}$. Notably, the top-left point $\left(D,C\right)=\left(\frac{10}{3},11\right)$ corresponds to the achievability scheme in [38], i.e., achieves the minimum possible download cost. On the other hand, the achievability of the point (D,C)=(4,5.5) is illustrated as a motivating example in Section 4.5 and Section 5.

By solving this optimization problem for a range of $D_{\mathrm{target}}$ values, we obtain a set of optimal operating points $\left(D_{\mathrm{target}},C_{\min}\left(D_{\mathrm{target}}\right)\right)$. Figure 2 presents such a storage–communication trade-off curve for a specific setting. It is crucial to note that this figure is plotted by numerically solving the optimization problem (21)–(24) via an exhaustive search-based strategy. In particular, for the specific system setting, we first discretize and sweep over a range of plausible values for the parameters $\eta_{1}$ and $\eta_{2}$ (subject to $\eta_{1},\eta_{2}\geq 1$). Then, for each candidate pair $\left(\eta_{1},\eta_{2}\right)$, we find achievable download cost vector $\left(D_{1},D_{2},\cdots ,D_{N}\right)$ that minimizes the total download cost $D_{\mathrm{target}}=\sum_{n}D_{n}$ for the specific η. For each feasible $\left(\eta_{1},\eta_{2}\right)$ and its associated optimal download vector $\left(D_{1},D_{2},\cdots ,D_{N}\right)$, the corresponding total storage cost $C=\sum_{n=1}^{N}C_{n}$ is calculated. This results in a point $\left(D_{\mathrm{target}},C\right)$ on the *D*-*C* plane for the given η. The set of all points $\left(D_{\mathrm{target}},C\right)$ obtained from all feasible $\left(\eta_{1},\eta_{2}\right)$ pairs forms a set of achievable $\left(D_{\mathrm{target}},C\right)$ points, and the trade-off curve is obtained by finding its lower convex hull. Although this numerical method does not guarantee the exploration of the entire feasible region, the resulting curve provides a conservative approximation of the optimal solution. In other words, the true optimal trade-off curve can only lie on or to the lower left of the curve presented in the figure, which serves as a bound for the $\left(D_{\mathrm{target}},C_{\min}\left(D_{\mathrm{target}}\right)\right)$ curve.

## 4. An Achievability Scheme for Asymmetric Setting

As discussed in the introduction, our achievability scheme construction begins with a scheme for Asymmetric MDS-GXSTPLC. Specifically, in this asymmetric variant, the security and privacy constraints are parameterized by two tuples $X=(X_{1},X_{2},\cdots ,X_{M})$ and $T=(T_{1},T_{2},\cdots ,T_{M})$, and for each message set m∈[M], the corresponding constraints are $X_{m}$-security and $T_{m}$-privacy, i.e.,

(25) $$\begin{array}{cccc} \; & I(S_{X};W_{m})=0, & \; & \forall X\subset \left[N\right],|X|=X_{m}, \end{array}$$

(26) $$\begin{array}{cccc} \; & I(Q_{T}^{\left(\Lambda \right)};{\left(\lambda_{m,k}\right)}_{k\in [K_{m}]})=0, & \; & \forall T\subset \left[N\right],|T|=T_{m}. \end{array}$$

Moreover, for each message set m∈[M], the codewords distributed among the servers $n\in R_{m}$ must form an $\left(N,X_{m}+\eta_{m}\right)$-MDS code; i.e., the messages $W_{m}$ must be recoverable from any $X_{m}+\eta_{m}$ of its codewords, and the size of any of the codeword is at most $L/\eta_{m}$ in *q*-ary units, where $\eta_{m}\in Z_{>0}$ for all m∈[M]. In particular, we show that the following (uniform) normalized download cost is achievable for Asymmetric MDS-GXSTPLC for all n∈[N]

(27) $$\begin{array}{c} D_{n}=\frac{1}{{\left(\min_{m\in [M]}\rho_{m}-X_{m}-T_{m}-\eta_{m}+1\right)}^{+}}. \end{array}$$

Once the achievability scheme for the asymmetric setting is established, we then employ the idea of the augmented system adapted from [38] to show that the normalized download cost in Theorem 1 is achievable. The remainder of this section is devoted to the presentation of the achievability scheme for the problem of Asymmetric MDS-GXSTPLC.

### 4.1. Preliminaries

Recall that the security and privacy level are parameterized by two tuples $X=(X_{1},X_{2},\cdots ,X_{M})$ and $T=(T_{1},T_{2},\cdots ,T_{M})$. Also, the MDS code constraint is parameterized by a set of *M* positive integers $\eta_{1},\eta_{2},\cdots ,\eta_{M}$. Moreover, let us assume that for all m∈[M], we have $\rho_{m}>X_{m}+T_{m}+\eta_{m}-1$; otherwise, our scheme is infeasible. Let us define $\mu =\min_{m\in [M]}\rho_{m}-X_{m}-T_{m}-\eta_{m}+1$, and set $L=\mathrm{lcm}(\mu ,\eta_{1},\eta_{2},\cdots ,\eta_{M})$; i.e., each of the *K* messages consists of *L* i.i.d. symbols from the finite field $F_{q}$. Recall that setting $L=\mathrm{lcm}(\mu ,\eta_{1},\eta_{2},\cdots ,\eta_{M})$ allows us to define a positive integer $J_{m}=L/\eta_{m}$ as we must have $\eta_{m}|L$. Now for all m∈[M], let us define a mapping $\phi_{m}:\left[J_{m}\right]\times \left[\eta_{m}\right]\to \left[L\right]$ as $\phi_{m}\left(\ell ,\kappa \right)=\ell +\left(\kappa -1\right)J_{m}$, i.e., the column-major order reshaping of [L]. It is obvious that $\phi_{m}$ is invertible, denoted as $\phi_{m}^{-1}$. Note that the mapping $\phi_{m}$ allows us to reshape message vectors ${\left(W_{m,k}\right)}_{k\in [K_{m}]}$ into $J_{m}\times \eta_{m}$ matrices for all m∈[M], where $J_{m}=L/\eta_{m}$. In other words, for all $m\in \left[M\right],k\in \left[K_{m}\right],\ell \in \left[J_{m}\right],\kappa \in \left[\eta_{m}\right]$, we define $W_{m,k}\left(\ell ,\kappa \right)=W_{m,k}\left(\phi_{m}\left(\ell ,\kappa \right)\right)$. Also, we need a total of N+L distinct elements from $F_{q}$, denoted as $\alpha_{1},\alpha_{2},\cdots ,\alpha_{N},f_{1},f_{2},\cdots ,f_{L}$. The existence of such distinct elements is guaranteed by selecting a sufficiently large field q≥N+L. For all $m\in \left[M\right],\ell \in \left[J_{m}\right],\kappa \in \left[\eta_{m}\right]$, let us define $f_{\ell ,\kappa }^{\left(m\right)}=f_{\phi_{m}\left(\ell ,\kappa \right)}$. Finally, for all $m\in \left[M\right],\ell \in \left[J_{m}\right],\kappa \in \left[\eta_{m}\right]$, let us define $W_{m,\ell ,\kappa }={\left[W_{m,1}\left(\ell ,\kappa \right),W_{m,2}\left(\ell ,\kappa \right),\cdots ,W_{m,K_{m}}\left(\ell ,\kappa \right)\right]}^{⊤}$ and for all m∈[M], define $\lambda_{m}={\left[\lambda_{m,1},\lambda_{m,2},\cdots ,\lambda_{m,K_{m}}\right]}^{⊤}$. Clearly, the desired linear combination can be equivalently written in the following form

(28) $$\begin{array}{c} \lambda_{\Lambda }\left(W\right)={\left(\sum_{m\in [M]}W_{m,\ell ,\kappa }^{⊤}\lambda_{m}\right)}_{\ell \in \left[J_{m}\right],\kappa \in \left[\eta_{m}\right]}. \end{array}$$

### 4.2. Construction of the Storage

For all m∈[M], let us define the following null-shaper polynomial in α

(29) $$\begin{array}{c} N_{m}\left(\alpha \right)=\prod_{n\in \left[N\right]\setminus R_{m}}\left(\alpha -\alpha_{n}\right), \end{array}$$

and for all $m\in \left[M\right],\ell \in \left[J_{m}\right]$, let us define the following (vector-valued) rational function in α

(30) $$\begin{array}{c} {\tilde{W}}_{m,\ell }\left(\alpha \right)=N_{m}\left(\alpha \right)\left(\sum_{\kappa \in [\eta_{m}]}\frac{N_{m}{\left(f_{\ell ,\kappa }^{\left(m\right)}\right)}^{-1}}{\alpha -f_{\ell ,\kappa }^{\left(m\right)}}W_{m,\ell ,\kappa }\;+\;\sum_{x\in [X_{m}]}\alpha^{x-1}Z_{m,\ell ,x}\right) \end{array}$$

where for all $m\in \left[M\right],\ell \in \left[J_{m}\right],x\in \left[X_{m}\right]$, $Z_{m,\ell ,x}$ are uniformly i.i.d. column vectors from $F_{q}^{K_{m}}$, independent of the messages. Note that by the definition of N(α), for all $m\in \left[M\right],n\in \left[N\right]\setminus R_{m}$, we have ${\tilde{W}}_{m,\ell }\left(\alpha_{n}\right)=0$, i.e., the rational function ${\tilde{W}}_{m,\ell }\left(\alpha \right)$, evaluated at the points corresponding to the servers that are prohibited from storing codewords of the $m^{th}$ message set, is zero. Moreover, by partial fraction decomposition, we can also write

(31) $$\begin{array}{cc} {\tilde{W}}_{m,\ell }\left(\alpha \right)= & \sum_{\kappa \in [\eta_{m}]}\frac{1}{\alpha -f_{\ell ,\kappa }^{\left(m\right)}}W_{m,\ell ,\kappa }+\sum_{i\in [X_{m}+N-\rho_{m}]}\alpha^{i-1}Y_{m,\ell ,i}, \end{array}$$

where for all $m\in \left[M\right],\ell \in \left[J_{m}\right]$, ${\left(Y_{m,\ell ,i}\right)}_{i\in [X_{m}+N-\rho_{m}]}$ are various linear combinations of ${\left(W_{m,\ell ,\kappa }\right)}_{\kappa \in [\eta_{m}]}$ and ${\left(Z_{m,\ell ,x}\right)}_{x\in [X_{m}]}$. Now, for all n∈[N], the storage at Server *n* is constructed as follows

(32) $$\begin{array}{c} S_{n}=\left\{{\tilde{W}}_{m,\ell }\left(\alpha_{n}\right)|m\in \left[M\right],\ell \in \left[J_{m}\right]\right\}. \end{array}$$

Again, while evaluating the rational function ${\tilde{W}}_{m,\ell }\left(\alpha \right)$ for all $\alpha_{n},n\in \left[N\right]$ might seem to violate the graph-based storage pattern, this paradox is resolved because our construction guarantees that for each message set m∈[M], the evaluations for servers that refrain from storing any codeword of that message set are explicitly set to zero; hence, no storage is necessary. Moreover, for all $m\in \left[M\right],k\in \left[K_{m}\right]$, the size of the codeword of $W_{m,k}$ for Server $n,n\in R_{m}$ is $J_{m}=L/\eta_{m}$. And for all $m\in \left[M\right],\ell \in \left[J_{m}\right]$ and $K\subset R_{m}$ such that $\left|K\right|=\eta_{m}+X_{m}$, given ${\left({\tilde{W}}_{m,\ell }\left(\alpha_{n}\right)\right)}_{n\in K}$, the message symbols ${\left(W_{m,\ell ,\kappa }\right)}_{\kappa \in [\eta_{m}]}$ are recoverable. This is because for all $n\in R_{m}$, we have $N(\alpha_{m})\neq 0$, so from ${\left(N_{m}{\left(\alpha_{m}\right)}^{-1}{\tilde{W}}_{m,\ell }\left(\alpha_{n}\right)\right)}_{n\in K}$, ${\left(W_{m,\ell ,\kappa }\right)}_{\kappa \in [\eta_{m}]}$ are recoverable by inverting the following (scaled) Cauchy–Vandermonde matrix

(33) $$\begin{array}{c} \left[\begin{array}{cccccc} \frac{N_{m}{\left(f_{\ell ,1}^{\left(m\right)}\right)}^{-1}}{\alpha_{i_{1}}-f_{\ell ,1}^{\left(m\right)}} & \cdots & \frac{N_{m}{\left(f_{\ell ,\eta_{m}}^{\left(m\right)}\right)}^{-1}}{\alpha_{i_{1}}-f_{\ell ,\eta_{m}}^{\left(m\right)}} & 1 & \cdots & \alpha_{i_{1}}^{X_{m}-1}\\ \vdots & \vdots & \vdots & \vdots & \vdots & \vdots \\ \frac{N_{m}{\left(f_{\ell ,1}^{\left(m\right)}\right)}^{-1}}{\alpha_{i_{\eta_{m}+X_{m}}}-f_{\ell ,1}^{\left(m\right)}} & \cdots & \frac{N_{m}{\left(f_{\ell ,\eta_{m}}^{\left(m\right)}\right)}^{-1}}{\alpha_{i_{\eta_{m}+X_{m}}}-f_{\ell ,\eta_{m}}^{\left(m\right)}} & 1 & \cdots & \alpha_{i_{\eta_{m}+X_{m}}}^{X_{m}-1} \end{array}\right] \end{array}$$

where $K=\{i_{1},i_{2},\cdots ,i_{\eta_{m}+X_{m}}\}$, and for any distinct $\alpha_{i_{1}},\alpha_{i_{2}},\cdots ,\alpha_{i_{\eta_{m}+X_{m}}},f_{\ell ,1}^{\left(m\right)},f_{\ell ,2}^{\left(m\right)},\cdots ,f_{\ell ,\eta_{m}}^{\left(m\right)}$, the Cauchy–Vandermonde matrix must be invertible. Finally, guaranteed by the $\left(N,X_{m}\right)$-MDS coded uniform noise terms, for all m∈[M], the storage at any $X_{m}$ servers is independent of $W_{m}$, i.e., the storage is $X_{m}$-secure with respect to the $m^{th}$ message set.

### 4.3. Construction of the Queries

For all $m\in \left[M\right],\ell \in \left[J_{m}\right],\kappa \in \left[\eta_{m}\right]$, let us define the following polynomial in α

(34) $$\begin{array}{cc} \; & Q_{m,\ell ,\kappa }\left(\alpha \right)=\prod_{\kappa^{\prime }\in \left[\eta_{m}\right]\setminus \left\{\kappa \right\}}\frac{\alpha -f_{\ell ,\kappa^{\prime }}^{\left(m\right)}}{f_{\ell ,\kappa }^{\left(m\right)}-f_{\ell ,\kappa^{\prime }}^{\left(m\right)}}\lambda_{m}+\prod_{\kappa^{\prime }\in \left[\eta_{m}\right]}\left(\alpha -f_{\ell ,\kappa^{\prime }}^{\left(m\right)}\right)\left(\sum_{t\in [T_{m}]}\alpha^{t-1}Z_{m,\ell ,\kappa ,t}^{\prime }\right), \end{array}$$

where for all $m\in \left[M\right],\ell \in \left[J_{m}\right],\kappa \in \left[\eta_{m}\right],t\in \left[T_{m}\right]$, $Z_{m,\ell ,\kappa ,t}^{\prime }$ are uniformly i.i.d. column vectors from $F_{q}^{K_{m}}$, independent of the coefficients. Now the query for Server *n* is constructed as

(35) $$\begin{array}{c} Q_{n}^{\left(\Lambda \right)}=\left\{Q_{m,\ell ,\kappa }\left(\alpha_{n}\right)|m\in \left[M\right],\ell \in \left[J_{m}\right],\kappa \in \left[\eta_{m}\right]\right\}. \end{array}$$

Similarly, due to the fact that the coefficients are protected by the $\left(N,T_{m}\right)$-MDS coded uniform noise terms, for all m∈[M], the queries are $T_{m}$-private with respect to the $m^{th}$ message set.

### 4.4. Construction of the Answers

Let us define V=L/μ, and note that μ∣L, *V* must be a positive integer. Once the query $Q_{n}^{\left(\Lambda \right)}$ is available at Server *n*, for all n∈[N], the answer $A_{n}^{\left(\Lambda \right)}$ is constructed as follows

(36) $$\begin{array}{cc} \; & A_{n}^{\left(\Lambda \right)}=\left\{\sum_{\begin{array}{c} m\in [M]\\ \left(\ell ,\kappa \right)\in \phi_{m}^{-1}\left[\left(v-1\right)\mu +1:v\mu \right] \end{array}}\;{\tilde{W}}_{m,\ell }{\left(\alpha_{n}\right)}^{⊤}Q_{m,\ell ,\kappa }\left(\alpha_{n}\right)\;|\;v\in \left[V\right]\right\}. \end{array}$$

To see the correctness of our scheme, note that the term ${\tilde{W}}_{m,\ell }{\left(\alpha_{n}\right)}^{⊤}Q_{m,\ell ,\kappa }\left(\alpha_{n}\right)$ can be viewed as the evaluation of the rational function ${\tilde{W}}_{m,\ell }{\left(\alpha \right)}^{⊤}Q_{m,\ell ,\kappa }\left(\alpha \right)$ at $\alpha_{n}$, where, exploiting the equivalent form of ${\tilde{W}}_{m,\ell }\left(\alpha \right)$ in (31), we can write

(37) $$\begin{array}{cc} \; & {\tilde{W}}_{m,\ell }{\left(\alpha \right)}^{⊤}Q_{m,\ell ,\kappa }\left(\alpha \right)=\frac{1}{\alpha -f_{\ell ,\kappa }^{\left(m\right)}}W_{m,\ell ,\kappa }^{⊤}\lambda_{m}+\sum_{i\in [X_{m}+N-\rho_{m}+\eta_{m}+T_{m}-1]}\alpha^{i-1}Y_{m,\ell ,\kappa ,i}^{\prime } \end{array}$$

where for all $m\in \left[M\right],\ell \in \left[J_{m}\right],\kappa \in \left[\eta_{m}\right],i\in \left[X_{m}+N-\rho_{m}+\eta_{m}+T_{m}-1\right]$, $Y_{m,\ell ,\kappa ,i}^{\prime }$ are various linear combinations of ${\left(W_{m,\ell ,\kappa }\right)}_{\kappa \in [\eta_{m}]}$, ${\left(Y_{m,\ell ,i}\right)}_{i\in [X_{m}+N-\rho_{m}]}$, $\lambda_{m}$ and ${\left(Z_{m,\ell ,\kappa ,t}^{\prime }\right)}_{t\in [T_{m}]}$. Note that by the definition of μ, for all m∈[M], we have $\mu \leq \rho_{m}-X_{m}-T_{m}-\eta_{m}+1$. Therefore $X_{m}+N-\rho_{m}+\eta_{m}+T_{m}-1=N-\left(\rho_{m}-X_{m}-T_{m}-\eta_{m}+1\right)\leq N-\mu$; i.e., we can equivalently write

(38) $$\begin{array}{cc} \; & {\tilde{W}}_{m,\ell }{\left(\alpha \right)}^{⊤}Q_{m,\ell ,\kappa }\left(\alpha \right)=\frac{1}{\alpha -f_{\ell ,\kappa }^{\left(m\right)}}W_{m,\ell ,\kappa }^{⊤}\lambda_{m}+\sum_{i\in [N-\mu ]}\alpha^{i-1}Y_{m,\ell ,\kappa ,i}^{\prime } \end{array}$$

where for all $N-(\rho_{m}-X_{m}-T_{m}-\eta_{m}+1)<i\leq N-\mu$, we simply set $Y_{m,\ell ,\kappa ,i}^{\prime }=0$. Now plug this form into the definition of the answer, and recall that for all m∈[M], $\phi_{m}$ represents the column-major order reshaping of [L], we have

(39) $$\begin{array}{cc} \; & \sum_{m\in \left[M\right],\left(\ell ,\kappa \right)\in \phi_{m}^{-1}\left[\left(v-1\right)\mu +1:v\mu \right]}{\tilde{W}}_{m,\ell }{\left(\alpha_{n}\right)}^{⊤}Q_{m,\ell ,\kappa }\left(\alpha_{n}\right)\\ \; & =\sum_{l\in [(v-1)\mu +1:v\mu ]}\frac{1}{\alpha_{n}-f_{l}}\left(\sum_{m\in [M]}W_{m,(\phi_{m}^{-1}\left(l\right))}^{⊤}\lambda_{m}\right)+\sum_{i\in [N-\mu ]}\alpha_{n}^{i-1}Y_{v,i}^{\prime \prime } \end{array}$$

where for all v∈[V],

(40) $$\begin{array}{c} Y_{v,i}^{\prime \prime }=\sum_{m\in \left[M\right],\left(\ell ,\kappa \right)\in \phi_{m}^{-1}\left[\left(v-1\right)\mu +1:v\mu \right]}Y_{m,\ell ,\kappa ,i}^{\prime }. \end{array}$$

Now it is clear that for all v∈[V], the desired linear combination symbols

${\left(\sum_{m\in [M]}W_{m,\ell ,\kappa }^{⊤}\lambda_{m}\right)}_{\left(\ell ,\kappa \right)\in \phi_{m}^{-1}\left[\left(v-1\right)\mu +1:v\mu \right]}$ can be decoded by inverting the following Cauchy–Vandermonde matrix

(41) $$\begin{array}{c} \underset{{CSA}_{N,\mu }^{q}\left(\alpha ,f\right)}{\underset{︸}{\left[\begin{array}{cccccc} \frac{1}{\alpha_{1}-f_{(v-1)\mu +1}} & \cdots & \frac{1}{\alpha_{1}-f_{v\mu }} & 1 & \cdots & \alpha_{1}^{N-\mu -1}\\ \vdots & \vdots & \vdots & \vdots & \vdots & \vdots \\ \frac{1}{\alpha_{N}-f_{(v-1)\mu +1}} & \cdots & \frac{1}{\alpha_{N}-f_{v\mu }} & 1 & \cdots & \alpha_{N}^{N-\mu -1} \end{array}\right]}}, \end{array}$$

whose invertibility is ensured by the fact that $f_{1},f_{2},\cdots ,f_{L},\alpha_{1},\alpha_{2},\cdots ,\alpha_{N}$ are distinct. Finally, note that for each server *n*, a total of *V* symbols are downloaded, and the normalized download cost of the scheme is, thus,

(42) $$\begin{array}{c} D_{n}=\frac{V}{L}=\frac{1}{\mu }=\frac{1}{\min_{m\in [M]}\rho_{m}-X_{m}-T_{m}-\eta_{m}+1},\forall n\in \left[N\right]. \end{array}$$

### 4.5. Motivating Example

Let us elaborate on our scheme via an illustrative example. Consider an example where we have N=20 servers that store M=2 message sets according to the following storage pattern.

(43a) $$\begin{array}{cccc} R_{1} & =\{3,4,5,6,7,8,13,14,15,16,17,18\},\;\;\; & \rho_{1} & =12, \end{array}$$

(43b) $$\begin{array}{cccc} R_{2} & =\{1,2,9,10,11,12,16,17,19,20\},\;\;\; & \rho_{2} & =10. \end{array}$$

Conversely, we can also write

(44) $$\begin{array}{c} \begin{array}{cccc} M_{1}=\left\{2\right\}, & M_{2}=\left\{2\right\}, & M_{3}=\left\{1\right\}, & M_{4}=\left\{1\right\},\\ M_{5}=\left\{1\right\}, & M_{6}=\left\{1\right\}, & M_{7}=\left\{1\right\}, & M_{8}=\left\{1\right\},\\ M_{9}=\left\{2\right\}, & M_{10}=\left\{2\right\}, & M_{11}=\left\{2\right\}, & M_{12}=\left\{2\right\},\\ M_{13}=\left\{1\right\}, & M_{14}=\left\{1\right\}, & M_{15}=\left\{1\right\}, & M_{16}=\left\{1\right\},\\ M_{17}=\left\{1\right\}, & M_{18}=\left\{1\right\}, & M_{19}=\left\{2\right\}, & M_{20}=\left\{2\right\}. \end{array} \end{array}$$

Moreover, let us set the asymmetric security and privacy thresholds $X_{1}=3,X_{2}=2$, $T_{1}=3,T_{2}=2$. Also, the MDS code constraint is parameterized by two positive integers $\eta_{1}=2,\eta_{2}=2$. According to the definition of Section 4.1, we have μ=5,L=10. Recall that the *K* messages are partitioned into two disjoint sets $W_{1}$ and $W_{2}$, where

(45a) $$\begin{array}{c} W_{1}=\left\{W_{1,1},W_{1,2},\cdots ,W_{1,K_{1}}\right\}, \end{array}$$

(45b) $$\begin{array}{c} W_{2}=\left\{W_{2,1},W_{2,2},\cdots ,W_{2,K_{2}}\right\}. \end{array}$$

Each message comprises L=10 symbols from $F_{q}$, where q≥30, i.e.,

(46) $$\begin{array}{cc} \; & W_{1,k}={\left[W_{1,k}\left(1\right),W_{1,k}\left(2\right),\cdots ,W_{1,k}\left(10\right)\right]}^{⊤},\;k\in \left[K_{1}\right], \end{array}$$

(47) $$\begin{array}{cc} \; & W_{2,k}={\left[W_{2,k}\left(1\right),W_{2,k}\left(2\right),\cdots ,W_{2,k}\left(10\right)\right]}^{⊤},\;k\in \left[K_{2}\right]. \end{array}$$

Let $\alpha_{1},\alpha_{2},\cdots ,\alpha_{20},f_{1,1},\cdots ,f_{5,1},f_{1,2},\cdots ,f_{5,2}$ be 30 distinct constants from the finite field $F_{q}$. According to the definition of Section 4.1, we have $J_{1}=L/\eta_{1}=5$ and $J_{2}=L/\eta_{2}=5$. For all ℓ∈[5],κ∈[2], let us define

(48) $$\begin{array}{cc} \; & W_{1,\ell ,\kappa }={\left[W_{1,1}\left(\ell +5\left(\kappa -1\right)\right),W_{1,2}\left(\ell +5\left(\kappa -1\right)\right),\cdots ,W_{1,K_{1}}\left(\ell +5\left(\kappa -1\right)\right)\right]}^{⊤} \end{array}$$

(49) $$\begin{array}{cc} \; & W_{2,\ell ,\kappa }={\left[W_{2,1}\left(\ell +5\left(\kappa -1\right)\right),W_{2,2}\left(\ell +5\left(\kappa -1\right)\right),\cdots ,W_{2,K_{2}}\left(\ell +5\left(\kappa -1\right)\right)\right]}^{⊤}. \end{array}$$

and define

(50) $$\begin{array}{cc} \; & \lambda_{1}={\left[\lambda_{1,1},\lambda_{1,2},\cdots ,\lambda_{1,K_{1}}\right]}^{⊤}, \end{array}$$

(51) $$\begin{array}{cc} \; & \lambda_{2}={\left[\lambda_{2,1},\lambda_{2,2},\cdots ,\lambda_{2,K_{2}}\right]}^{⊤}. \end{array}$$

Then the desired linear combination of the two messages can be written in the following form.

(52) $$\begin{array}{c} \lambda_{\Lambda }\left(W\right)={\left[\sum_{m\in [2]}W_{m,1,1}^{⊤}\lambda_{m},\cdots ,\sum_{m\in [2]}W_{m,5,1}^{⊤}\lambda_{m},\sum_{m\in [2]}W_{m,1,2}^{⊤}\lambda_{m},\cdots ,\sum_{m\in [2]}W_{m,5,2}^{⊤}\lambda_{m}\right]}^{⊤}. \end{array}$$

For all m∈[2], let us define the following null-shaper polynomial in α,

(53) $$\begin{array}{c} N_{m}\left(\alpha \right)=\prod_{n\in \left[N\right]\setminus R_{m}}\left(\alpha -\alpha_{n}\right). \end{array}$$

Let us define the following (vector-valued) rational function in α,

(54) $$\begin{array}{cc} {\tilde{W}}_{1,\ell }\left(\alpha \right)≜ & N_{1}\left(\alpha \right)\left(\frac{N_{1}{\left(f_{\ell ,1}\right)}^{-1}}{\alpha -f_{\ell ,1}}W_{1,\ell ,1}+\frac{N_{1}{\left(f_{\ell ,2}\right)}^{-1}}{\alpha -f_{\ell ,2}}W_{1,\ell ,2}+\sum_{x\in [3]}\alpha^{x-1}Z_{1,\ell ,x}\right) \end{array}$$

(55) $$\begin{array}{cc} = & \frac{1}{\alpha -f_{\ell ,1}}W_{1,\ell ,1}+\frac{1}{\alpha -f_{\ell ,2}}W_{1,\ell ,2}+\sum_{i\in [11]}\alpha^{i-1}Y_{1,\ell ,i}. \end{array}$$

Note that since for all $n\in \left[N\right]\setminus R_{1}$, $N_{1}\left(\alpha_{n}\right)=0$, we have ${\tilde{W}}_{1,\ell }\left(\alpha_{n}\right)=0$ for all ℓ∈[5]. In (55), for all ℓ∈[5], ${\left(Y_{1,\ell ,i}\right)}_{i\in [11]}$ are various linear combinations of ${\left(W_{1,\ell ,\kappa }\right)}_{\kappa \in [2]}$ and ${\left(Z_{1,\ell ,x}\right)}_{x\in [3]}$. Since $|R_{1}|=12$, the degree of the last term (viewed as a polynomial in α) in (55) is 10.

Let us define the following (vector-valued) rational function in α,

(56) $$\begin{array}{cc} {\tilde{W}}_{2,\ell }\left(\alpha \right) & ≜N_{2}\left(\alpha \right)\left(\frac{N_{2}{\left(f_{\ell ,1}\right)}^{-1}}{\alpha -f_{\ell ,1}}W_{2,\ell ,1}+\frac{N_{2}{\left(f_{\ell ,2}\right)}^{-1}}{\alpha -f_{\ell ,2}}W_{2,\ell ,2}+\sum_{x\in [2]}\alpha^{x-1}Z_{2,\ell ,x}\right) \end{array}$$

(57) $$\begin{array}{cc} \; & =\frac{1}{\alpha -f_{\ell ,1}}W_{2,\ell ,1}+\frac{1}{\alpha -f_{\ell ,2}}W_{2,\ell ,2}+\sum_{i\in [12]}\alpha^{i-1}Y_{2,\ell ,i} \end{array}$$

Note that since for all $n\in \left[N\right]\setminus R_{2}$, $N_{2}\left(\alpha_{n}\right)=0$, we have ${\tilde{W}}_{2,\ell }\left(\alpha_{n}\right)=0$ for all ℓ∈[5]. In (57), for all ℓ∈[5], ${\left(Y_{2,\ell ,i}\right)}_{i\in [12]}$ are various linear combinations of ${\left(W_{2,\ell ,\kappa }\right)}_{\kappa \in [2]}$ and ${\left(Z_{2,\ell ,x}\right)}_{x\in [2]}$. Since $|R_{2}|=10$, the degree of the last term (viewed as a polynomial in α) in (57) is 11.

For all n∈[20], the storage at Server *n* is $S_{n}=\left\{{\tilde{W}}_{1,\ell }\left(\alpha_{n}\right),{\tilde{W}}_{2,\ell }\left(\alpha_{n}\right)|\ell \in \left[5\right]\right\}$. The MDS(20,3) coded random noise term in (54) guarantees that the messages ${\left(W_{1,k}\right)}_{k\in [K_{1}]}$ is $X_{1}=3$-secure. Similarly, the MDS(20,2) coded random noise term in (56) guarantees that the message ${\left(W_{2,k}\right)}_{k\in [K_{2}]}$ is $X_{2}=2$-secure. Thus we have

(58a) $$\begin{array}{cccc} \; & I(S_{X};W_{1})=0, & \; & \forall X\subset [20],|X|=3, \end{array}$$

(58b) $$\begin{array}{cccc} \; & I(S_{X};W_{2})=0, & \; & \forall X\subset [20],|X|=2. \end{array}$$

Note that for all $n\in \left[20\right],m\in \left[2\right]\setminus M_{n},\ell \in \left[5\right]$, ${\tilde{W}}_{m,\ell }\left(\alpha_{n}\right)=0$ holds, i.e., if the graph-based storage pattern prohibits a server from storing a certain message, the corresponding codeword is explicitly set to zero.

For all ℓ∈[5],κ∈[2],t∈[3], let $Z_{1,\ell ,\kappa ,t}^{\prime }$ be uniformly i.i.d. column vectors from $F_{q}^{K_{1}}$, independent of the coefficients. For all ℓ∈[5], let us define the following rational functions in α,

(59) $$\begin{array}{cc} \; & Q_{1,\ell ,1}\left(\alpha \right)=\frac{\alpha -f_{\ell ,2}}{f_{\ell ,1}-f_{\ell ,2}}\lambda_{1}+\left(\alpha -f_{\ell ,1}\right)\left(\alpha -f_{\ell ,2}\right)\left(\sum_{t\in [3]}\alpha^{t-1}Z_{1,\ell ,1,t}^{\prime }\right), \end{array}$$

(60) $$\begin{array}{cc} \; & Q_{1,\ell ,2}\left(\alpha \right)=\frac{\alpha -f_{\ell ,1}}{f_{\ell ,2}-f_{\ell ,1}}\lambda_{1}+\left(\alpha -f_{\ell ,1}\right)\left(\alpha -f_{\ell ,2}\right)\left(\sum_{t\in [3]}\alpha^{t-1}Z_{1,\ell ,2,t}^{\prime }\right), \end{array}$$

For all ℓ∈[5],κ∈[2],t∈[2], let $Z_{2,\ell ,\kappa ,t}^{\prime }$ be uniformly i.i.d. column vectors from $F_{q}^{K_{2}}$, independent of the coefficients. For all ℓ∈[5], let us define the following rational functions in α,

(61) $$\begin{array}{cc} \; & Q_{2,\ell ,1}\left(\alpha \right)=\frac{\alpha -f_{\ell ,2}}{f_{\ell ,1}-f_{\ell ,2}}\lambda_{2}+\left(\alpha -f_{\ell ,1}\right)\left(\alpha -f_{\ell ,2}\right)\left(\sum_{t\in [2]}\alpha^{t-1}Z_{2,\ell ,1,t}^{\prime }\right), \end{array}$$

(62) $$\begin{array}{cc} \; & Q_{2,\ell ,2}\left(\alpha \right)=\frac{\alpha -f_{\ell ,1}}{f_{\ell ,2}-f_{\ell ,1}}\lambda_{2}+\left(\alpha -f_{\ell ,1}\right)\left(\alpha -f_{\ell ,2}\right)\left(\sum_{t\in [2]}\alpha^{t-1}Z_{2,\ell ,2,t}^{\prime }\right), \end{array}$$

For all n∈[20], the query sent to Server *n* is constructed as $Q_{n}^{\left(\Lambda \right)}=\{Q_{1,\ell ,\kappa }\left(\alpha_{n}\right),$$Q_{2,\ell ,\kappa }\left(\alpha_{n}\right)|\ell \in \left[5\right],\kappa \in \left[2\right]\}$. The MDS(20,3) coded random noise terms in (59) and (60) guarantee that the coefficient $\lambda_{1}$ is $T_{1}=3$–private. Similarly, the MDS(20,2) coded random noise terms in (61) and (62) guarantee that the coefficient $\lambda_{2}$ is $T_{2}=2$-private. Thus we have

(63a) $$\begin{array}{cccc} \; & I(Q_{T}^{\left(\Lambda \right)};{\left(\lambda_{1,k}\right)}_{k\in [K_{1}]})=0, & \; & \forall T\subset [20],|T|=3, \end{array}$$

(63b) $$\begin{array}{cccc} \; & I(Q_{T}^{\left(\Lambda \right)};{\left(\lambda_{2,k}\right)}_{k\in [K_{2}]})=0, & \; & \forall T\subset [20],|T|=2. \end{array}$$

According to the definition of Section 4.1, V=L/μ=2. The answer returned by Server *n*, n∈[20] is constructed as follows.

(64) $$\begin{array}{c} A_{n}^{\left(\Lambda \right)}=\left\{\sum_{m\in [2],\ell \in [5]}{\tilde{W}}_{m,\ell }{\left(\alpha_{n}\right)}^{⊤}Q_{m,\ell ,1}\left(\alpha_{n}\right),\sum_{m\in [2],\ell \in [5]}{\tilde{W}}_{m,\ell }{\left(\alpha_{n}\right)}^{⊤}Q_{m,\ell ,2}\left(\alpha_{n}\right),\right\}, \end{array}$$

where

$$\begin{array}{cc} \; & \sum_{m\in [2],\ell \in [5]}{\tilde{W}}_{m,\ell }{\left(\alpha_{n}\right)}^{⊤}Q_{m,\ell ,1}\left(\alpha_{n}\right)\\ = & \sum_{\ell \in [5]}{\left(\frac{1}{\alpha_{n}-f_{\ell ,1}}W_{1,\ell ,1}+\frac{1}{\alpha_{n}-f_{\ell ,2}}W_{1,\ell ,2}+\sum_{i\in [11]}\alpha_{n}^{i-1}Y_{1,\ell ,i}\right)}^{⊤}\\ \; & \;\times \left[\frac{\alpha_{n}-f_{\ell ,2}}{f_{\ell ,1}-f_{\ell ,2}}\lambda_{1}+\left(\alpha_{n}-f_{\ell ,1}\right)\left(\alpha_{n}-f_{\ell ,2}\right)\left(\sum_{t\in [3]}\alpha_{n}^{t-1}Z_{1,\ell ,1,t}^{\prime }\right)\right]\\ \; & \;+{\left(\frac{1}{\alpha_{n}-f_{\ell ,1}}W_{2,\ell ,1}+\frac{1}{\alpha_{n}-f_{\ell ,2}}W_{2,\ell ,2}+\sum_{i\in [12]}\alpha_{n}^{i-1}Y_{2,\ell ,i}\right)}^{⊤} \end{array}$$

(65) $$\begin{array}{c} \;\times \left[\frac{\alpha_{n}-f_{\ell ,2}}{f_{\ell ,1}-f_{\ell ,2}}\lambda_{2}+\left(\alpha_{n}-f_{\ell ,1}\right)\left(\alpha_{n}-f_{\ell ,2}\right)\left(\sum_{t\in [2]}\alpha_{n}^{t-1}Z_{2,\ell ,1,t}^{\prime }\right)\right] \end{array}$$

(66) $$\begin{array}{cc} = & \sum_{\ell \in [5]}\left(\frac{1}{\alpha_{n}-f_{\ell ,1}}W_{1,\ell ,1}^{⊤}\lambda_{1}+\frac{1}{\alpha_{n}-f_{\ell ,1}}W_{2,\ell ,1}^{⊤}\lambda_{2}+\sum_{s\in [15]}\alpha_{n}^{s-1}Y_{\ell ,1,s}^{\prime }\right) \end{array}$$

(67) $$\begin{array}{cc} = & \sum_{\ell \in [5]}\frac{1}{\alpha_{n}-f_{\ell ,1}}\left(\sum_{m\in [2]}W_{m,\ell ,1}^{⊤}\lambda_{m}\right)+\sum_{s\in [15]}\alpha_{n}^{s-1}{\bar{Y}}_{1,s} \end{array}$$

where for all ℓ∈[5],s∈[15], $Y_{\ell ,1,s}^{\prime }$ are various linear combinations of $W_{1,\ell ,2}^{⊤}\lambda_{1}$, ${\left(Y_{1,\ell ,i}^{⊤}\lambda_{1}\right)}_{i\in [11]}$, ${\left(W_{1,\ell ,2}^{⊤}Z_{1,\ell ,1,t}^{\prime }\right)}_{t\in [3]}$, ${\left(Y_{1,\ell ,i}^{⊤}Z_{1,\ell ,1,t}^{\prime }\right)}_{t\in [3],i\in [12]}$, $W_{2,\ell ,2}^{⊤}\lambda_{2}$, ${\left(Y_{2,\ell ,i}^{⊤}\lambda_{2}\right)}_{i\in [12]}$, ${\left(W_{2,\ell ,2}^{⊤}Z_{2,\ell ,1,t}^{\prime }\right)}_{t\in [2]}$, ${\left(Y_{2,\ell ,i}^{⊤}Z_{2,\ell ,1,t}^{\prime }\right)}_{t\in [2],i\in [12]}$, whose exact form is not relevant. Similarly, we have

(68) $$\begin{array}{cc} \; & \sum_{m\in [2],\ell \in [5]}{\tilde{W}}_{m,\ell }{\left(\alpha_{n}\right)}^{⊤}Q_{m,\ell ,2}\left(\alpha_{n}\right)\\ = & \sum_{\ell \in [5]}\frac{1}{\alpha_{n}-f_{\ell ,2}}\left(\sum_{m\in [2]}W_{m,\ell ,2}^{⊤}\lambda_{m}\right)+\sum_{s\in [15]}\alpha_{n}^{s-1}{\bar{Y}}_{2,s}. \end{array}$$

Note that for all $n\in \left[20\right],m\in \left[2\right]\setminus M_{n},\ell \in \left[5\right]$, ${\tilde{W}}_{m,\ell }\left(\alpha_{n}\right)=0$ always holds; thus, there is no need for the user to upload $Q_{m,\ell ,1}$ and $Q_{m,\ell ,2}$.

Now, we can write the symbols contained in ${\left(A_{n}^{\left(\Lambda \right)}\right)}_{n\in [20]}$ in the following matrix form,

(69) $$\begin{array}{cc} \; & \left[\begin{array}{c} \sum_{m\in [2],\ell \in [5]}{\tilde{W}}_{m,\ell }{\left(\alpha_{1}\right)}^{⊤}Q_{m,\ell ,1}\left(\alpha_{1}\right)\\ \sum_{m\in [2],\ell \in [5]}{\tilde{W}}_{m,\ell }{\left(\alpha_{2}\right)}^{⊤}Q_{m,\ell ,1}\left(\alpha_{2}\right)\\ \vdots \\ \sum_{m\in [2],\ell \in [5]}{\tilde{W}}_{m,\ell }{\left(\alpha_{20}\right)}^{⊤}Q_{m,\ell ,1}\left(\alpha_{20}\right) \end{array}\right]\\ = & \left[\begin{array}{ccccccc} \frac{1}{\alpha_{1}-f_{1,1}} & \cdots & \frac{1}{\alpha_{1}-f_{5,1}} & 1 & \alpha_{1} & \cdots & \alpha_{1}^{13}\\ \frac{1}{\alpha_{2}-f_{1,1}} & \cdots & \frac{1}{\alpha_{2}-f_{5,1}} & 1 & \alpha_{2} & \cdots & \alpha_{2}^{13}\\ \vdots \\ \frac{1}{\alpha_{20}-f_{1,1}} & \cdots & \frac{1}{\alpha_{20}-f_{5,1}} & 1 & \alpha_{20} & \cdots & \alpha_{20}^{13} \end{array}\right]\\ \; & \times {\left[\sum_{m\in [2]}W_{m,1,1}^{⊤}\lambda_{m}\;\cdots \;\sum_{m\in [2]}W_{m,5,1}^{⊤}\lambda_{m}\;{\bar{Y}}_{1,1}\;{\bar{Y}}_{1,2}\;\cdots \;{\bar{Y}}_{1,15}\right]}^{⊤} \end{array}$$

(70) $$\begin{array}{cc} \; & \left[\begin{array}{c} \sum_{m\in [2],\ell \in [5]}{\tilde{W}}_{m,\ell }{\left(\alpha_{1}\right)}^{⊤}Q_{m,\ell ,2}\left(\alpha_{1}\right)\\ \sum_{m\in [2],\ell \in [5]}{\tilde{W}}_{m,\ell }{\left(\alpha_{2}\right)}^{⊤}Q_{m,\ell ,2}\left(\alpha_{2}\right)\\ \vdots \\ \sum_{m\in [2],\ell \in [5]}{\tilde{W}}_{m,\ell }{\left(\alpha_{20}\right)}^{⊤}Q_{m,\ell ,2}\left(\alpha_{20}\right) \end{array}\right]\\ = & \left[\begin{array}{ccccccc} \frac{1}{\alpha_{1}-f_{1,2}} & \cdots & \frac{1}{\alpha_{1}-f_{5,2}} & 1 & \alpha_{1} & \cdots & \alpha_{1}^{13}\\ \frac{1}{\alpha_{2}-f_{1,2}} & \cdots & \frac{1}{\alpha_{2}-f_{5,2}} & 1 & \alpha_{2} & \cdots & \alpha_{2}^{13}\\ \vdots \\ \frac{1}{\alpha_{20}-f_{1,2}} & \cdots & \frac{1}{\alpha_{20}-f_{5,2}} & 1 & \alpha_{20} & \cdots & \alpha_{20}^{13} \end{array}\right]\\ \; & \times {\left[\sum_{m\in [2]}W_{m,\ell ,2}^{⊤}\lambda_{m}\;\cdots \;\sum_{m\in [2]}W_{m,\ell ,2}^{⊤}\lambda_{m}\;{\bar{Y}}_{2,1}\;{\bar{Y}}_{2,2}\;\cdots \;{\bar{Y}}_{2,15}\right]}^{⊤} \end{array}$$

Now it is clear that the desired linear combination symbols ${\left[\sum_{m\in [M]}W_{m,\ell ,\kappa }^{⊤}\lambda_{m}\right]}_{\ell \in [5],\kappa \in [2]}$ can be decoded by inverting the Cauchy–Vandermonde matrix in (69) and (70), respectively, whose invertibility is ensured by the fact that $f_{1,1},\cdots ,f_{5,1},f_{1,2},\cdots ,f_{5,2},\alpha_{1},\alpha_{2},\cdots ,\alpha_{20}$ are distinct. Finally, note that for each server *n*, a total of two symbols are downloaded, and the normalized download cost of the scheme is, thus, $D_{n}=2/10=1/5$ for all n∈[20].

**Remark** **2.** *It is of interest to compare the decoding complexity of our scheme with that of [38]. For the decoding procedure, our scheme directly inverts a Cauchy–Vandermonde matrix, which can be conducted in $O(N^{3})$ via standard methods or $\tilde{O}\left(N\log^{2}N\right)$ using fast algorithms. In contrast, the scheme in [38] requires an additional pre-processing step for interference cancellation before a similar inversion, incurring an extra complexity of $O(N^{2})$ above the complexity of inverting a Vandermonde matrix (which is also $O(N^{3})$ via standard methods or $\tilde{O}\left(N\log^{2}N\right)$ using fast algorithms).*

## 5. Proof of Theorem 1

In this section, adapting from the idea of the augmented system in [38], we show that the download cost and the corresponding storage cost as defined in Theorem 1 are achievable. The augmented system is an Asymmetric MDS-GXSTPLC instance with the same message sets that are to be eventually reduced to the original GXSTPLC setting by merging servers. Specifically, the augmented system consists of $\bar{N}=\sum_{n\in [N]}\tau_{n}$ servers, denoted as Server $\left(1,1\right),\left(1,2\right),\cdots ,\left(1,\tau_{1}\right),\cdots ,\left(N,1\right),\left(N,2\right),\cdots ,\left(N,\tau_{N}\right)$. The storage pattern $\bar{R}=\left\{{\bar{R}}_{1},{\bar{R}}_{2},\cdots ,{\bar{R}}_{M}\right\}$ and security/privacy thresholds for each message set $\bar{X}=\left({\bar{X}}_{1},{\bar{X}}_{2},\cdots ,{\bar{X}}_{M}\right)$, $\bar{T}=\left({\bar{T}}_{1},,{\bar{T}}_{2},\cdots ,{\bar{T}}_{M}\right)$ are defined as follows.

(71) $$\begin{array}{cc} {\bar{X}}_{m} & =X\gamma_{m},\;\forall m\in \left[M\right] \end{array}$$

(72) $$\begin{array}{cc} {\bar{T}}_{m} & =T\gamma_{m},\;\forall m\in \left[M\right] \end{array}$$

(73) $$\begin{array}{cc} {\bar{R}}_{m} & =\left\{\left(n,i\right)|n\in R_{m},i\in \left[\min\left(\gamma_{m},\tau_{n}\right)\right]\right\} \end{array}$$

In addition, for all m∈[M], we set the MDS coding parameter for the augmented system ${\bar{\eta }}_{m}=q_{0}\left(\eta_{m}-1\right)+1$. Recall that by the definition of $q_{0}$, ${\bar{\eta }}_{m}$ must be an integer.

Our GXSTPLC scheme is obtained by merging servers $\left(n,1\right),\left(n,2\right),\cdots ,\left(n,\tau_{n}\right)$ into Server *n* for all n∈[N], i.e., assigning the storage, queries, and corresponding answers. Note that this recovers the original storage pattern R because for all n∈[N], we have ${⋃}_{i\in [\tau_{n}]}{\bar{M}}_{\left(n,i\right)}\subseteq M_{n}$, where ${\bar{M}}_{\left(n,i\right)}=\left\{m\in \left[M\right]|{\bar{R}}_{m}∋\left(n,i\right)\right\}$ is the dual representation of $\bar{R}$. Moreover, due to the fact that for all m∈[M], $n\in R_{m}$, the total number of pairs of the form (n,∗) in ${\bar{R}}_{m}$ cannot be greater than ${\bar{X}}_{m}/X$ and ${\bar{T}}_{m}/T$, so any *X* (or *T*) colluding servers have, at most, ${\bar{X}}_{m}$ (or ${\bar{T}}_{m}$) codewords (or queries) of the $m^{th}$ message set. Guaranteed by the $X_{m}$-security (or $T_{m}$-privacy), these colluding servers disclose nothing about the messages (or coefficients); i.e., the scheme is *X*-secure and *T*-private. Finally, according to Section 4, for the augmented system, the normalized download cost of

(74) $$\begin{array}{c} D_{n,i}=\frac{1}{\min_{m\in [M]}{\bar{\rho }}_{m}-{\bar{X}}_{m}-{\bar{T}}_{m}-{\bar{\eta }}_{m}+1} \end{array}$$

for each server $\left(n,i\right),n\in \left[\bar{N}\right],i\in \left[\tau_{n}\right]$ is achievable, where ${\bar{\rho }}_{m}=\left|{\bar{R}}_{m}\right|,m\in \left[M\right]$. According to the definition, for all m∈[M], ${\bar{\rho }}_{m}={\bar{X}}_{m}+{\bar{T}}_{m}+\nu_{m}$, where $\nu_{m}$ is the summation of the smallest $\left(\rho_{m}-X-T\right)$ elements in ${\left(\tau_{n}\right)}_{n\in R_{m}}$. Since $(D_{1},D_{2},\cdots ,D_{N})\in D$, we must have $\nu_{m}\geq q_{0}\eta_{m}$ for all m∈[M]. Now, since reducing the augmented system to the original setting does not increase the download cost, we can verify that the desired normalized download cost $D_{n}$ for all Server n,n∈[N] is achievable by our scheme, as follows.

(75) $$\begin{array}{cc} \sum_{i\in [\tau_{n}]}D_{n,i}= & \frac{\tau_{n}}{\min_{m\in [M]}{\bar{\rho }}_{m}-{\bar{X}}_{m}-{\bar{T}}_{m}-{\bar{\eta }}_{m}+1} \end{array}$$

(76) $$\begin{array}{cc} = & \frac{\tau_{n}}{\min_{m\in [M]}\nu_{m}-{\bar{\eta }}_{m}+1} \end{array}$$

(77) $$\begin{array}{cc} \leq & \frac{\tau_{n}}{q_{0}\eta_{m}-q_{0}\left(\eta_{m}-1\right)} \end{array}$$

(78) $$\begin{array}{cc} = & \frac{\tau_{n}}{q_{0}} \end{array}$$

(79) $$\begin{array}{cc} = & D_{n} \end{array}$$

Moreover, the normalized storage cost of Server *n*, $C_{n}$, can be calculated as follows.

(80) $$\begin{array}{cc} C_{n} & =\frac{\sum_{i\in \left[\tau_{n}\right],m\in {\bar{M}}_{n,i}}K_{m}/{\bar{\eta }}_{m}}{K} \end{array}$$

(81) $$\begin{array}{cc} \; & =\frac{1}{K}\sum_{i=1}^{\tau_{n}}\sum_{m=1}^{M}I\left(\left(n,i\right)\in {\bar{R}}_{m}\right)\frac{K_{m}}{{\bar{\eta }}_{m}} \end{array}$$

(82) $$\begin{array}{cc} \; & =\frac{1}{K}\sum_{m\in M_{n}}\min\left(\tau_{n},\gamma_{m}\right)\frac{K_{m}}{q_{0}\left(\eta_{m}-1\right)+1} \end{array}$$

**Remark** **3.** *Recall that our achievability scheme is constructed by reducing the augmented system (i.e., an Asymmetric MDS-GXSTPLC instance) to the original setting. Consequently, the decoding procedure for the scheme consists of inverting a series of Cauchy–Vandermonde matrices defined in (41). Indeed, this can be viewed as a series of instances of the CSA scheme, where the desired linear combination symbols are carried by the Cauchy terms, and the interference symbols are aligned within the Vandermonde terms. Then, according to the N-Sum Box abstraction of CSA codes [44,50], in a quantum setting where servers can send entangled qudits via separate quantum channels to the user, representing encoded classical answer symbols through local quantum operations, the superdense coding gain is achievable. In particular, for any total normalized download cost of $D=\sum_{n\in [N]}D_{n}>2$ in the classical setting, the corresponding quantum scheme achieves a total download cost of $\frac{D}{2}$. For any classical total normalized download cost of D<2, the quantum scheme achieves the (obviously) optimal normalized total download cost of D=1. Note that this direct application of the CSA structure and the subsequent quantum gain is not directly applicable to schemes exploiting properties of dual GRS codes [26,38], since their decoding requires a pre-processing procedure of interference cancellation, and the resulting structure does not follow that of CSA codes.*

### Motivating Example

Consider another motivating example where we have N=8 servers and *K* messages, X=1 and T=1. The *K* messages are partitioned into two disjoint sets $W_{1}$ and $W_{2}$. Let us set $K_{1}=K_{2}=K/2$. The storage pattern for this example is as follows.

(83a) $$\begin{array}{cc} R_{1} & =\{2,3,6,7\}, \end{array}$$

(83b) $$\begin{array}{cc} R_{2} & =\{1,4,5,7,8\}. \end{array}$$

Let us set $\eta =\left(\eta_{1},\eta_{2}\right)=\left(1.2,1.2\right)$. Moreover, let us also set the target vector of per-server normalized download costs as $\left(D_{1},D_{2},\cdots ,D_{8}\right)=\left(2/5,3/5,3/5,2/5,2/5,3/5,3/5,2/5\right)$. It can be easily verified that the selected vector of per-server normalized download costs lies in the feasible region D(η) defined in (15). Following the notations defined in Theorem 1 and above, we have $q_{0}=5$, $\left(\tau_{1},\tau_{2},\cdots ,\tau_{6}\right)=\left(2,3,3,2,2,3,3,2\right)$ and $\bar{N}=20$. The 20 servers in the augmented system are listed as ((1,1),(1,2),(2,1),(2,2),(2,3),(3,1),(3,2),(3,3),(4,1),(4,2),(5,1),(5,2),(6,1),(6,2),(6,3),(7,1),(7,2),(7,3),(8,1),(8,2)).

Now we can generate the augmented system. Specifically, for the augmented system, we have ${\bar{X}}_{3}={\bar{T}}_{1}=3$, ${\bar{X}}_{2}={\bar{T}}_{2}=2$, ${\bar{\eta }}_{1}={\bar{\eta }}_{2}=2$, and the storage pattern is shown in Table 1.

This is exactly the setting presented in the motivating example in Section 4.5 with server mapping Ψ:((1,1),(1,2),(2,1),(2,2),(2,3),(3,1),(3,2),(3,3),(4,1),(4,2),(5,1),(5,2),(6,1),(6,2),(6,3),(7,1),(7,2),(7,3),(8,1),(8,2))→(1,2,⋯,20), and for each server $\left(n,i\right),n\in \left[8\right],i\in \left[\tau_{n}\right]$, the normalized download cost of $D_{n,i}=1/5$ is achievable. After reducing the augmented system to the original setting, we can calculate that the normalized download costs $\left(D_{1},D_{2},\cdots ,D_{8}\right)=\left(2/5,3/5,3/5,2/5,2/5,3/5,3/5,2/5\right)$ are achievable, and the normalized storage costs are $\left(C_{1},C_{2},\cdots ,C_{8}\right)=\left(1/2,3/4,3/4,1/2,1/2,3/4,5/4,1/2\right)$.

Now let us see why the resulting GXSTPLC scheme is X=1–secure and T=1–private. Note that for all n∈{2,3,6},

(84) $$\begin{array}{cc} I(S_{n};W)= & I\left(\left\{{\tilde{W}}_{1,\ell }\left(\alpha_{\Psi ((n,i))}\right),{\tilde{W}}_{2,\ell }\left(\alpha_{\Psi ((n,i))}\right)|i\in \left[3\right],\ell \in \left[5\right]\right\};W_{1},W_{2}\right) \end{array}$$

(85) $$\begin{array}{cc} = & I\left(\left\{{\tilde{W}}_{1,\ell }\left(\alpha_{\Psi ((n,i))}\right)|i\in \left[3\right],\ell \in \left[5\right]\right\};W_{1}\right) \end{array}$$

(86) $$\begin{array}{cc} = & 0, \end{array}$$

(87) $$\begin{array}{ccc} I(Q_{n}^{\left[\Lambda \right]};\Lambda )= & I(\{Q_{1,\ell ,\kappa }\left(\alpha_{\Psi ((n,i))}\right),Q_{2,\ell ,\kappa }\left(\alpha_{\Psi ((n,i))}\right)|i\in \left[3\right],\ell \in \left[5\right],\kappa \in \left[2\right]\};\; & {\left\{\lambda_{m,k}\right\}}_{m\in \left[2\right],k\in \left[K_{m}\right]}) \end{array}$$

(88) $$\begin{array}{cc} = & I\left(\left\{Q_{1,\ell ,\kappa }\left(\alpha_{\Psi ((n,i))}\right)|i\in \left[3\right],\ell \in \left[5\right],\kappa \in \left[2\right]\right\};{\left\{\lambda_{1,k}\right\}}_{k\in [K_{1}]}\right) \end{array}$$

(89) $$\begin{array}{cc} = & 0, \end{array}$$

For all n∈{1,4,5,8},

(90) $$\begin{array}{cc} I(S_{n};W)= & I\left(\left\{{\tilde{W}}_{1,\ell }\left(\alpha_{\Psi ((n,i))}\right),{\tilde{W}}_{2,\ell }\left(\alpha_{\Psi ((n,i))}\right)|i\in \left[2\right],\ell \in \left[5\right]\right\};W_{1},W_{2}\right) \end{array}$$

(91) $$\begin{array}{cc} = & I\left(\left\{{\tilde{W}}_{2,\ell }\left(\alpha_{\Psi ((n,i))}\right)|i\in \left[2\right],\ell \in \left[5\right]\right\};W_{2}\right) \end{array}$$

(92) $$\begin{array}{cc} = & 0, \end{array}$$

(93) $$\begin{array}{ccc} I(Q_{n}^{\left[\Lambda \right]};\Lambda )= & I(\{Q_{1,\ell ,\kappa }\left(\alpha_{\Psi ((n,i))}\right),Q_{2,\ell ,\kappa }\left(\alpha_{\Psi ((n,i))}\right)|i\in \left[2\right],\ell \in \left[5\right],\kappa \in \left[2\right]\};\; & {\left\{\lambda_{m,k}\right\}}_{m\in \left[2\right],k\in \left[K_{m}\right]}) \end{array}$$

(94) $$\begin{array}{cc} = & I\left(\left\{Q_{2,\ell ,\kappa }\left(\alpha_{\Psi ((n,i))}\right)|i\in \left[2\right],\ell \in \left[5\right],\kappa \in \left[2\right]\right\};{\left\{\lambda_{2,k}\right\}}_{k\in [K_{2}]}\right) \end{array}$$

(95) $$\begin{array}{cc} = & 0, \end{array}$$

and

(96) $$\begin{array}{cc} I(S_{7};W)= & I\left(\left\{{\tilde{W}}_{1,\ell }\left(\alpha_{\Psi ((7,i))}\right),{\tilde{W}}_{2,\ell }\left(\alpha_{\Psi ((7,i))}\right)|i\in \left[3\right],\ell \in \left[5\right]\right\};W_{1},W_{2}\right)\\ = & I\left(\left\{{\tilde{W}}_{1,\ell }\left(\alpha_{\Psi ((7,i))}\right)|i\in \left[3\right],\ell \in \left[5\right]\right\};W_{1}\right) \end{array}$$

(97) $$\begin{array}{cc} \; & +I\left(\left\{{\tilde{W}}_{2,\ell }\left(\alpha_{\Psi ((7,i))}\right)|i\in \left[2\right],\ell \in \left[5\right]\right\};W_{2}\right) \end{array}$$

(98) $$\begin{array}{cc} = & 0, \end{array}$$

(99) $$\begin{array}{ccc} I(Q_{7}^{\left[\Lambda \right]};\Lambda )= & I(\{Q_{1,\ell ,\kappa }\left(\alpha_{\Psi ((7,i))}\right),Q_{2,\ell ,\kappa }\left(\alpha_{\Psi ((7,i))}\right)|i\in \left[3\right],\ell \in \left[5\right],\kappa \in \left[2\right]\};\; & {\left\{\lambda_{m,k}\right\}}_{m\in \left[2\right],k\in \left[K_{m}\right]})\\ = & I\left(\left\{Q_{1,\ell ,\kappa }\left(\alpha_{\Psi ((7,i))}\right)|i\in \left[3\right],\ell \in \left[5\right],\kappa \in \left[2\right]\right\};{\left\{\lambda_{1,k}\right\}}_{k\in [K_{1}]}\right) \end{array}$$

(100) $$\begin{array}{cc} \; & +I(\left\{Q_{2,\ell ,\kappa }\left(\alpha_{\Psi ((7,i))}\right)|i\in \left[2\right],\ell \in \left[5\right],\kappa \in \left[2\right]\right\};{\left\{\lambda_{2,k}\right\}}_{k\in [K_{2}]}) \end{array}$$

(101) $$\begin{array}{cc} = & 0, \end{array}$$

(86), (92), and (98) hold due to (58), (89), (95) and (101) holds due to (63).

## 6. Conclusions

We explored the problem of GXSTPLC by proposing an achievability scheme that establishes a trade-off between communication cost and storage cost. Notably, our scheme demonstrates the generalization of the CSA null shaper idea beyond its application in storage-consistent private updates. The application of CSA null shaper preserves the standard CSA decoding structure while offering significant advantages, including reduced decoding complexity and a direct framework for quantum transformation. Promising avenues for future research include the complete capacity characterization for the MDS-GXSTPLC problem. Additionally, investigating the potential applicability of the CSA null shaper to other relevant problems is of considerable interest.

## Figures and Tables

**Table 1:** Storage pattern of the augmented system in Example 1.

Server	(1,1)	(1,2)	(2,1)	(2,2)	(2,3)	(3,1)	(3,2)	(3,3)	(4,1)
${\bar{M}}_{\left(n,i\right)}$	2	2	1	1	1	1	1	1	2
								
Server	(4,2)	(5,1)	(5,2)	(6,1)	(6,2)	(6,3)	(7,1)	(7,2)	(7,3)
${\bar{M}}_{\left(n,i\right)}$	2	2	2	1	1	1	1	1	1
							2	2	
Server	(8,1)	(8,2)							
${\bar{M}}_{\left(n,i\right)}$	2	2							
								

## Data Availability

The data are contained within the article.

## Abbreviations

The following abbreviations are used in this manuscript:

CSA	Cross-Subspace Alignment
GXSTPLC	Graph-Based X-Secure T-Private Linear Computation
GRS	Generalized Reed–Solomon
PIR	Private Information Retrieval
PLC	Private Linear Computation
